# The Influence of Non Polar and Polar Molecules in Mouse Motile Cells Membranes and Pure Lipid Bilayers

**DOI:** 10.1371/journal.pone.0059364

**Published:** 2013-04-02

**Authors:** Francisco J. Sierra-Valdez, Linda S. Forero-Quintero, Patricio A. Zapata-Morin, Miguel Costas, Arturo Chavez-Reyes, Jesús C. Ruiz-Suárez

**Affiliations:** 1 CINVESTAV-Monterrey, Apodaca, Nuevo León, México; 2 Departamento de Fisicoquímica, Facultad de Química, Universidad Nacional Autónoma de México, Cd. Universitaria, México D.F., México; King's College London, United Kingdom

## Abstract

We report an experimental study of mouse sperm motility that shows chief aspects characteristic of neurons: the anesthetic (produced by tetracaine) and excitatory (produced by either caffeine or calcium) effects and their antagonic action. While tetracaine inhibits sperm motility and caffeine has an excitatory action, the combination of these two substances balance the effects, producing a motility quite similar to that of control cells. We also study the effects of these agents (anesthetic and excitatory) on the melting points of pure lipid liposomes constituted by 1,2-dipalmitoyl-sn-glycero-3-phosphocholine (DPPC) and dipalmitoyl phosphatidic acid (DPPA). Tetracaine induces a large fluidization of the membrane, shifting the liposomes melting transition temperature to much lower values. The effect of caffeine is null, but its addition to tetracaine-doped liposomes greatly screen the fluidization effect. A high calcium concentration stiffens pure lipid membranes and strongly reduces the effect of tetracaine. Molecular Dynamics Simulations are performed to further understand our experimental findings at the molecular level. We find a strong correlation between the effect of antagonic molecules that could explain how the mechanical properties suitable for normal cell functioning are affected and recovered.

## Introduction

Hundreds of molecules produce anesthesia, but only two models explain how they inhibit nerve impulses [1]–[3]. The oldest and most succinct model, hints toward a thermodynamic explanation. It states that anesthetic molecules act in the lipids medium of neuron cells and that the more hydrophobic such molecules are, the higher their narcotic potency. The newest and more accepted model, professes that the narcotic action occurs over specific binding sites of membrane proteins. And even if this second framework requires a diligent search of the binding sites for the innumerable anesthetic molecules, research and support on the protein paradigm is overwhelming. Meanwhile, the old rule, which states that the anesthetic potency is proportional to anesthetic solubility in membranes [4], remains infallible: no anesthetic molecule exists, whatsoever, with a partition coefficient less than one.

Recently, a very appealing idea able to explain the mechanical, thermal and electrical phenomena encountered during the nerve impulse has been proposed by T. Heimburg and A. D. Jackson [5], in which they propose a general model based on the existence of solitons as the main actors of the nerve impulse. This idea gives a coherent explanation on the excitability of membranes [6], [7], their permeability changes [8], reversible heat processes [9], [10] and general anesthesia, which, in turn, is supported by a simple thermodynamic explanation of the physical chemical effect of freezing-point depression [11]. Moreover, Heimburg and Jackson's idea provides an immediate and intuitive explanation for the reversal of anesthesia with pressure, temperature, calcium and pH; reversibility that is not easily explained, as far as we know, by the protein model. Indeed, it is hard to explain why the effect of an anesthetic, normally seen as an induced blockage of the molecular structure of ion channels, is reversed by an hydrostatic pressure (for example, it has been reported that the swimming motion of tadpoles disappears when they are anesthetized with ethanol, but the motion reappears when the anesthetized tadpoles are exposed to a hydrostatic pressure of 13–35 MPa [12]).

In addition to nerve cells, there are motile cells with a strong dependence on the elastic properties of the plasma membrane; one being the spermatozoon. Current theories suggest that a certain elastic condition is a prerequisite for normal functioning, and that the membrane fluidity and flexibility are mainly dependent on both their lipid constitution and the thermodynamic conditions, such as temperature [13]. So, inasmuch as in these cells the thermodynamic stability of the plasma membrane plays a crucial role in their motility, and following the idea that all cells should be affected by anesthetics at some doses, sperm motility is a good model to study the effect produced by such drugs. Hence, despite seem heretical, given the dominance of the ion channel picture [11], there are a variety of reasons for considering a macroscopic thermodynamic view for the general anesthesia based on lipid properties.

In this article we aim to advance Heimburg's et al model in the context of cellular motility. In the literature, some works explain sperm motility based on membrane proteins functioning. For instance, it is proposed that two calcium channels are related to sperm motility, in which the calcium ion plays an important role [14], [15]. Furthermore, many studies have been conducted with sperm under the effect of various anesthetic drugs, for example, tetracaine, lidocaine, diltiazem, and even some gases as sevoflurane and isoflurane, which due to its inhibitory action on cell motility, have been speculated as calcium channel blockers [16]–[18]. On the other hand, excitatory drugs in neurons, such as caffeine, have been shown as stimulator of human sperm motility [19]. Different mechanisms have been proposed based on a specific role of the cyclic amino monophosphate cAMP [20]. However, some other studies contradict such mechanisms [21], [22], so the arguments remain insufficient. Other study in which caffeine is antagonist of the inhibitory effects of procaine and propranolol was reported in [23] without any further explanation. The study of the caffeine interaction with pure lipid membranes has not been done, while some studies have shown that tetracaine strongly perturb the lipid bilayer system and change its phase behavior and thermomechanical properties [24], [25].

We explore the narcotic effect of tetracaine, a well known local antesthetic molecule, in the motility of mouse sperm and thereafter, study the antagonic effects produced by either caffeine or calcium. We then carry out a Differential Scanning Calorimetry (DSC) study of protein-free liposomes constituted by 1,2-dipalmitoyl-sn-glycero-3-phosphocholine (DPPC) and dipalmitoyl phosphatidic acid (DPPA) doped with tetracaine, caffeine and calcium. Finally, in order to obtain a molecular picture of the action of these drugs, we performed Molecular Dynamics Simulations of the lipid-caffeine-tetracaine system. Altogether, both experimental and numerical results suggest that polar agents screen the effect produced by non-polar molecules. Since plasma membranes are composed by lipids, we speculate that the same phenomenon occurs in any living cell.

## Results

### Sperm motility

**Figure 1:**
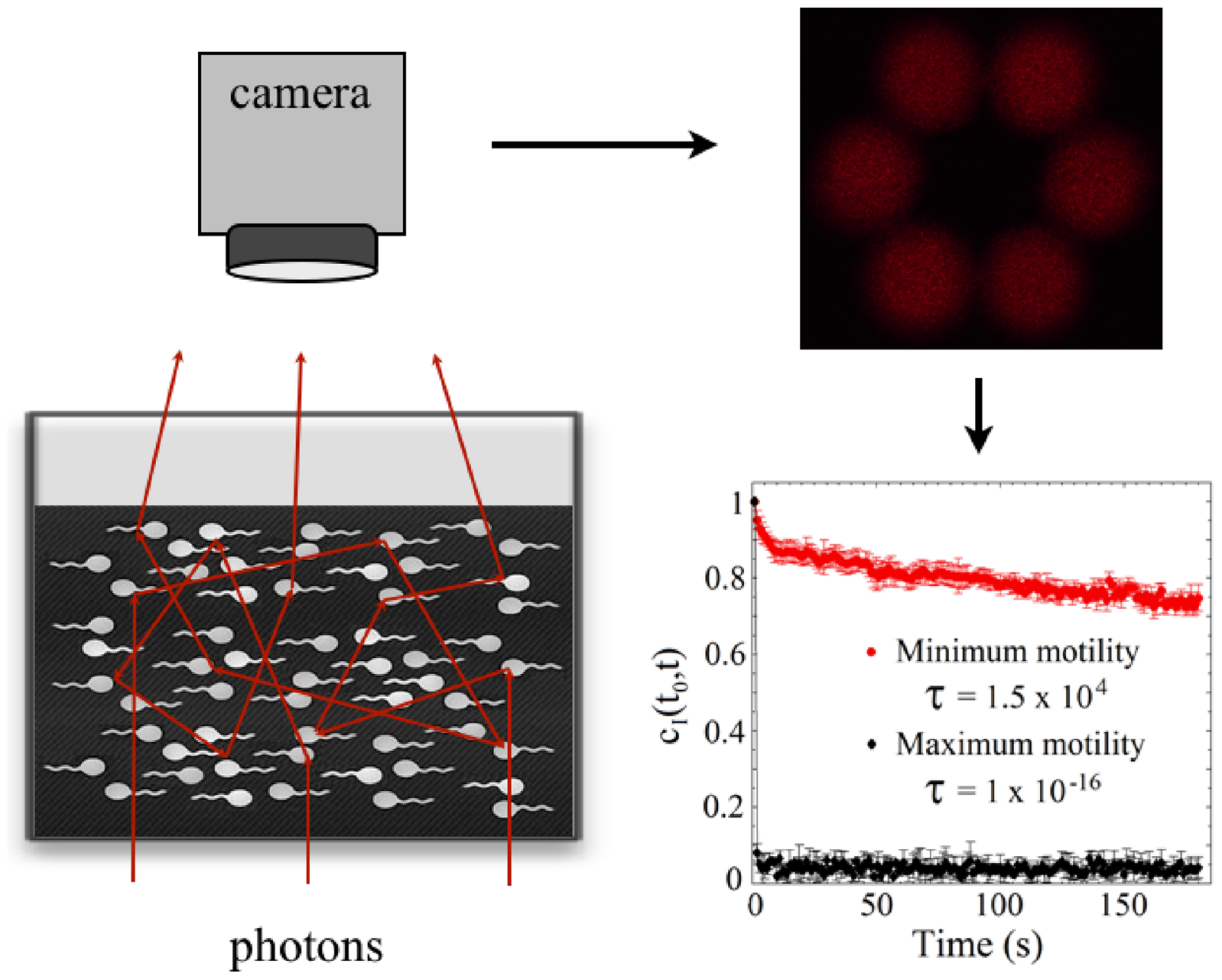
Schematic drawing of the cell motility sensor. A beam of a He-Ne laser is optically driven to a chamber that contains a sample holder. This sample holder has six small holes that can be filled with the sample. Light passes through each one of these receptacles. The scattering pattern is recorded by a digital camera for Image Correlation Analysis. See details in [26].

The motility studies were carried out by using a cell motility sensor recently developed by us [26] and schematically shown in Figure 1. It is based on an optical technique with a time-resolved correlation adapted in a system to handle several samples simultaneously, under controlled conditions. Sperm motility information is analysed through the temporal development of the cell motility parameter (CMP) 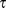, which increases as the motility decreases [26]. The loose of sperm motility with time can be related to several factors: a) inherent metabolism of the cell; b) pressure and/or temperature; c) drugs. However, not always the loose of motility is due to cellular death. For example, see the effect of thermal reversibility in [26].

**Figure 2:**
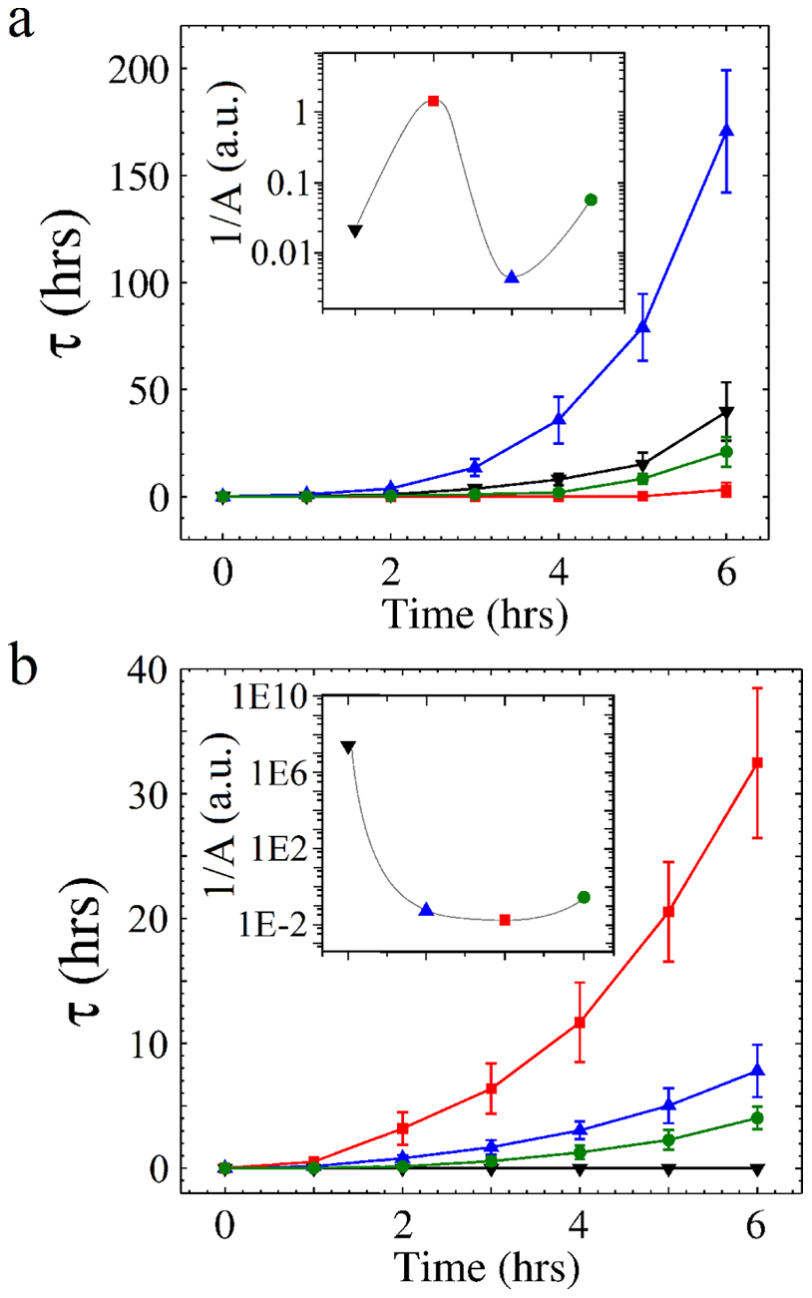
Sperm motility analysis: Temporal behavior of 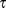. (a) Tetracaine-caffeine experiment (25°C): Triangles down mark the control sample, squares mark 111 mM of caffeine, triangles up mark 8 mM of tetracaine, and circles mark the combination 8 mM of tetracaine-111 mM of caffeine. (b) Tetracaine-calcium experiment (10°C): Triangles down mark the control sample, triangles up 8 mM of tetracaine, squares 200 mM of CaCl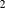 and circles the combination 8 mM of tetracaine-200 mM of CaCl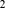. Insets show the inverse of the area under curve (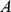) of each one of the temporal developments, highlighting the differences in overall motility.

Figure 2 illustrates the temporal behavior of 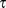 during 6 hours in the tetracaine-caffeine experiment at 25°C, where there is a noticeable contrast between the rapid inhibition of motility produced by tetracaine alone (i.e. cells are anesthetized) and the excitatory action of caffeine seen as a higher prevalence of motility. However, the combination of these two substances balance each other, producing a motility similar to the control cells. In Figure 2b, we now show a similar temporal behavior of 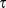 in the tetracaine-calcium experiment at 10°C: a high calcium concentration induces a strong inhibition of the cell motility (even more than tetracaine does), but the combination of both partially counteract each other and the prevalence of cell motility is improved. Since sperm motility is strongly temperature dependent [26], the controls of Figure 2 are different.

### Calorimetric study

DPPC/DPPA liposomes show a relatively high melting temperature of 42.5°C, so the energy invested to bring the membrane to the fluid state is greater than for many other lipid membranes.

**Figure 3:**
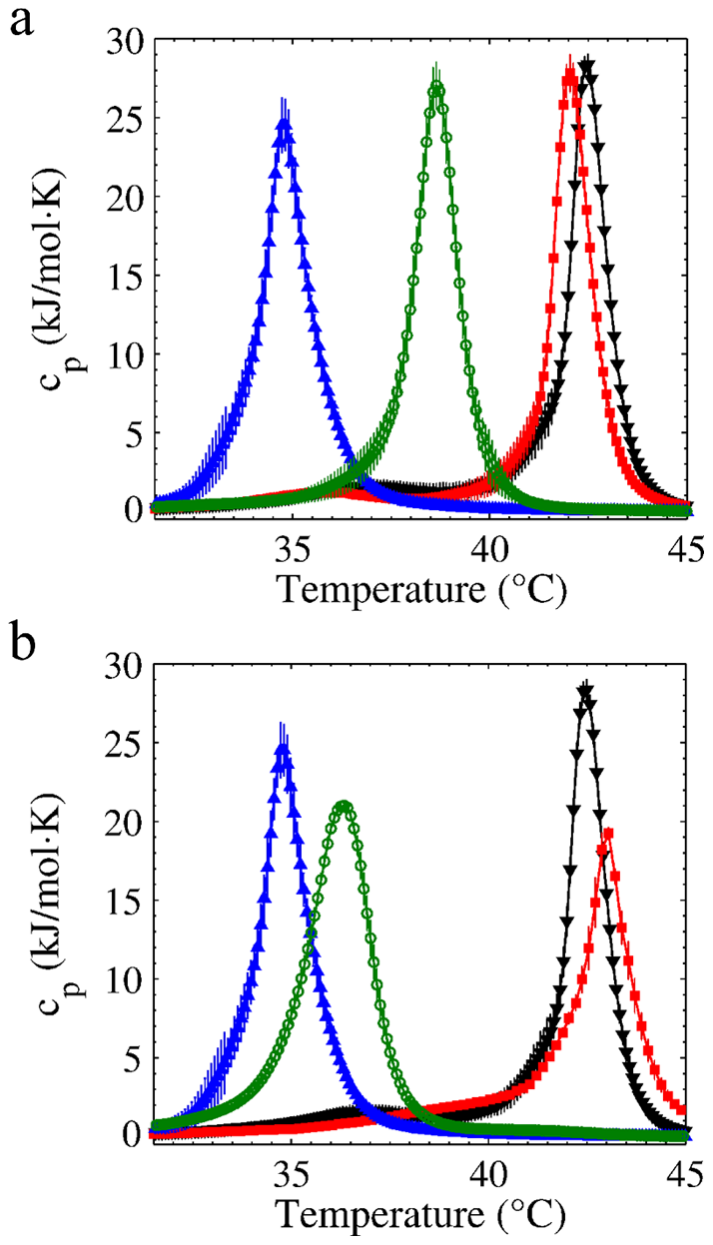
Calorimetric study of DPPC/DPPA liposomes. (a) Tetracaine-caffeine experiment: Triangles down are for the control sample, triangles up for 8 mM of tetracaine, squares for 111 mM of caffeine and circles for the combination 8 mM tetracaine-111 mM of caffeine. (b) Tetracaine-calcium experiment: triangles down are for the control sample, triangles up for 8 mM of tetracaine, squares for 200 mM of CaCl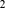 and circles for the combination 8 mM tetracaine-200 mM of CaCl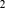.

The hydrophobic regions of the caffeine molecule are able to slightly fluidize the rigid DPPC/DPPA system, as indicated by the small shift to lower temperatures in Figure 3a. The same figure also displays how 8 mM of tetracaine induces a larger shift in the melting transition of the liposomes to lower temperatures. It is remarkable that the addition of caffeine, that acting alone has the ability to slightly weaken the rigid DPPC/DPPA liposomes, greatly rigidizes the tetracaine-doped ones.

**Figure 4:**
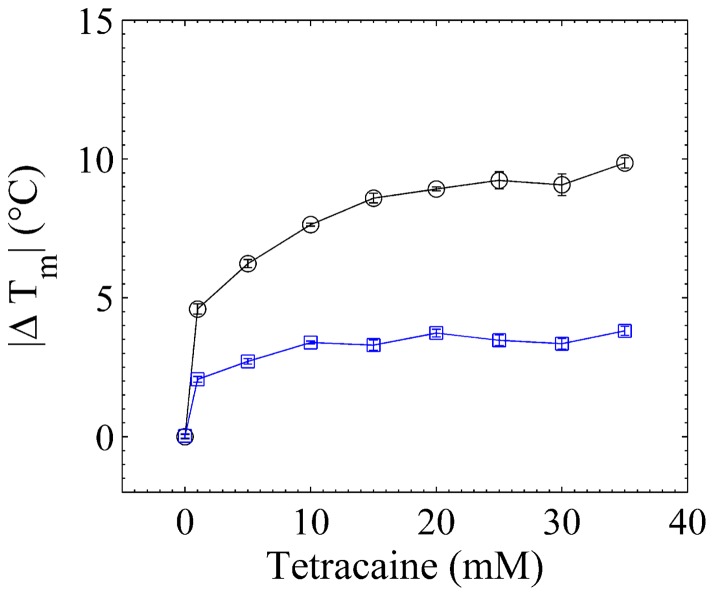
Calorimetric study of DPPC/DPPA liposomes. Tetracaine-caffeine experiment for several doses of tetracaine: shift of the melting temperature with respect to the control sample of the tetracaine doped liposomes at various doses with (squares) and without (circles) 111 mM of caffeine. Since 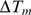 is always negative (the final temperature is always lower than the initial one), we plot its absolute number to illustrate the saturation of the shift at high concentrations of tetracaine. Note that, at the doses reported in this work, 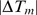 exhibits a Michaelis-Menten like behavior in both cases.

In other words, caffeine produces a slight disorder when the membrane is well compacted, but induces order when the membrane is not. The effect of caffeine on higher doses of tetracaine is even more pronounced as depicted in Figure 4. Remarkably, a pure polar screening agent (i.e. calcium) does not display the dichotomy produced by caffeine. In Figure 3b we show the calorimetric profiles of the tetracaine-calcium experiment, where a high calcium concentration always increases (slightly or greatly) the stability of (pure or tetracaine doped) liposomes.

### Molecular Dynamics Simulations

**Figure 5:**
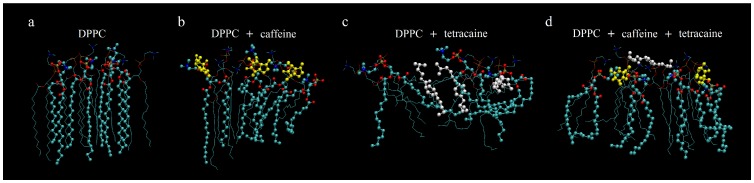
Molecular Dynamic snapshots of a DPPC/drugs system. (a) Control membrane showing a high ordered system. Red and blue dots highlight the polar region of the lipids (oxygen and nitrogen, respectively), chains of light blue dots the hydrophobic tails. (b) Caffeine (yellow) interacts mostly with the hydrophilic heads. (c) Tetracaine (white) inserts inside the membrane disrupting visibly the lipids organization at the hydrophobic region. (d) The combination of caffeine and tetracaine induces less disorder. The snapshots correspond to positions obtained 15 ns after the start of the simulation.

**Figure 6:**
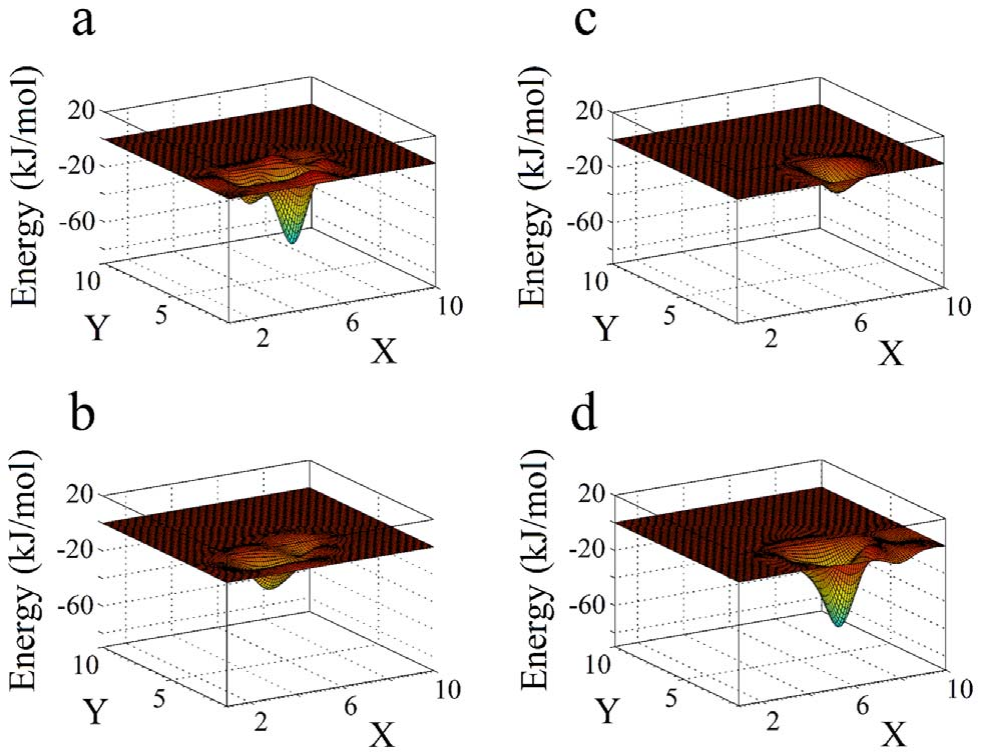
Representative energy landscapes. These are constructed by taking the interaction of a single caffeine and a single tetracaine molecule with each lipid of the upper membrane surface (an array of 10×10 lipids). Each landscape shows the morphology of the deepest potential well of the drug/membrane system simulation. (a) Coulomb potential for caffeine. (b) Lennard-Jones potential for caffeine. (c) Coulomb potential for tetracaine. (d) Lennard-Jones potential for tetracaine. The scales in the X–Y planes are in lipid units (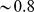 nm).

**Figure 7:**
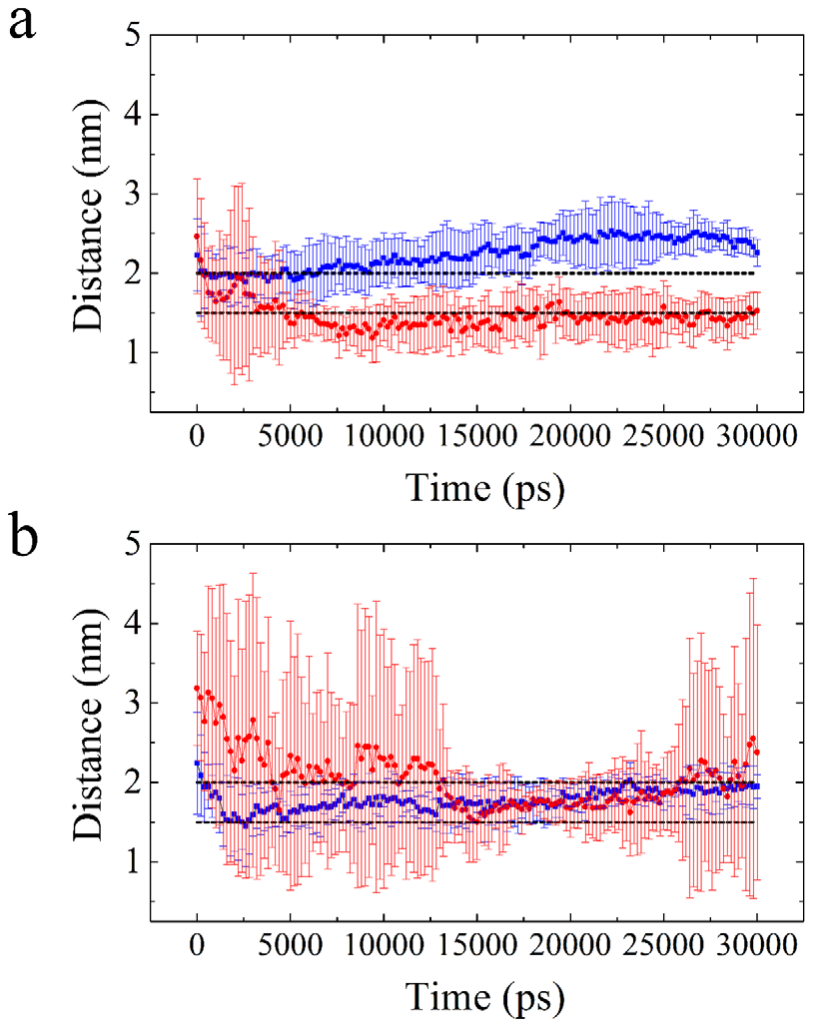
Full average trajectories obtained by Molecular Dynamic Simulations. (a) shows the average trajectories of caffeine (blue squares) and tetracaine (red dots) at independent simulations (ten molecules for each case) and (b) shows the average trajectories of both molecules in the same simulation (five and five). The dashed lines delimit the polar head lipid region of the upper membrane surface. The error bars are calculated by considering the trajectories of all the molecules used in the simulations. See details in the text.

Figures 5 and 6 display images obtained by Molecular Dynamics Simulations (MDS), showing, respectively, a small region of the upper part of DPPC membranes and the specific interaction sites with caffeine, tetracaine and caffeine plus tetracaine, and a landscape of the Coulomb and van der Waals interaction energies. Figure 7 shows the complete drug trajectories obtained by MDS (each point represents the average of the trajectories of the center of mass of the molecules).

## Discussion

When a membrane is at a temperature 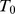 lower than its melting transition 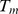, any agent that shifts 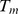 to lower temperatures increases the compressibility 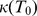 (the effect is maximum when 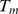 reaches 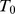), whilst a shift of 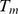 to higher temperatures stands for a decrease in 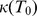 [27]. This course of events helps us to understand the effect an anesthetic produces.

In lipid membranes, there is a permanent electrostatic repulsion between the electric dipole heads (polar region) of the lipids, which are in a favourable interaction with water molecules. Despite this repulsion (somewhat screened by the presence of water and ions), the Van der Waals forces existing between the tails of the lipid (hydrophobic region) are large enough to produce the structure of the membrane effectively excluding water from its lipid region. Now, the high electric polarizability of tetracaine (31.379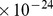 cm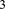) [28] responsible for its high hydrophobicity and partition coefficient, in addition to its molecular structure (see Figure 5c), are such that, once it inserts into the membrane, weakens the Van der Waals interactions, and thereby disturbing the organization of the membrane. We would like to remark that this phenomenon arises from the interplay between enthalpy and entropy. When the drug inserts between two lipid tails, it reduces their pairwise cohesive interaction, possibly by an Axilrod-Teller three-body potential that most of the time is positive [29]. Therefore, the solid-fluid transition shifts to lower temperatures, see Figure 3a. This fact allows us to consider the possibility that the fluidization of the sperm plasma membrane might be responsible for the decrease of cell motility in Figure 2a. It is clear that while pure tetracaine induces disorder (liposomes) or motility decrease (sperm cells) the inclusion of caffeine reverts the effect. We also observe in Figure 4 that the more the membrane is fluidized with tetracaine, the larger the compensatory effect of caffeine.

Even if thermodynamics offers a clear interpretation of the system studied here, a molecular dynamic picture is needed. Therefore, the above reasoning may gain in strength with the aid of Molecular Dynamic Simulations, which are useful to explore the specific molecular interactions that take place in a drug-membrane system.

The snapshot depicted in Figure 5 shows that caffeine, with its high polar nature that interacts with the heads of the lipids, produces a minor disarray compared to the one produced by tetracaine, which penetrates into the amphiphilic region. Indeed, Figure 6 shows how caffeine and tetracaine interact with the lipids of the membrane when they are at the surface. Coulomb interaction is greater for caffeine (see Figure 6a), and Lennard-Jones greater for tetracaine (see Figure 6d), explaining why the first only interacts with the polar heads of the lipids while the second molecule inserts itself into the amphiphilic region of the membrane (as shown in Figure 5). Moreover, when both caffeine and tetracaine are added together, due to its large-range Coulomb interaction, caffeine reaches the membrane faster than tetracaine. We speculate that the caffeine molecules increase the packing of the polar heads of the lipids, preventing tetracaine molecules to insert beyond the polar region of the membrane and fluidize it. Such behavior is clearly observed in the trajectories of both molecules in Figure 7b.

Protons and any other positive ions like calcium are also capable of producing a screening effect at the negative heads of the lipids, giving rise to a significant decrease in the electrostatic repulsion. In fact, some studies mention that Ca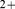 binds to phospholipids via a two-point attachment to adjacent phospholipid head groups [30], and melting points of such screened lipid membranes are increased [27].

In summary, both calcium and caffeine independently increase the sperm motility at 25°C, while they decrease it at 10°C. The reason is that the sperm motility is optimum at 10°C (sperm has the optimum elastic properties of the plasma membrane [26]). So, caffeine and calcium recover (by rigidization) the fluidized membrane at 25°C, while they over rigidize it at 10°C. As in other cells, the motion of the sperm flagella is due to molecular motors. Nevertheless, as mentioned above, the normal functioning of sperm cells is strongly dependent on the elastic properties of the plasma membrane [13], thereby any disturbance on such optimal properties would be reflected as a change in cell motility. This idea, in addition to our motility/calorimetric results, allow us to suggest that sperm plasma membrane transition temperature would be affected in the same way by these antagonic drugs.

The mouse sperm motility alteration and the doped protein-free liposomes calorimetric response, induced by tetracaine, indicate that any plasma membrane might be similarly perturbed. We propose an alternative model that does not need to resort to the existence and action of membrane proteins to explain the antagonic effect of caffeine and calcium against anesthesia. Following the steps of T. Heimburg and A. D. Jackson [5], this model could be extrapolated to nerve cells based only on the physical properties of the plasma membranes, in particular the compressibility and its effect in the soliton velocity.

## Materials and Methods

### Sperm sample preparation

The sperm were recovered from male mice CD1 aged 3–5 months. Mice were sacrificed by cervical dislocation and epididymis were dissected and rinsed in 2 mL of a Whitten-Hepes (WH) medium which comprises (in mM): 135 NaCl, 5 KCl, 2 CaCl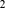, 1 MgSO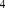, 10 HEPES, 10 glucose and 1 sodium pyruvate at pH 7.3 (NaOH). The WH medium keeps the physiological conditions for sperm incubation [31]. Several incisions were made in the epididymis in the solution and a swim-up method was used to separate sperm with greater than 90% of motility [32]. Finally, the upper part of the solution which contains the more motile sperm is isolated in another container in order to avoid contamination of epididymal tissues, thus, getting a final sperm suspension. Further, to avoid bacterial growth, penicillin and streptomycin are used as antibiotics, in a concentration of 1% for each sample. A careful study was performed to ensure that antibiotics do not affect sperm motility at this concentration. More details about the isolation, preparation and motility analysis of the sperm sample are described in [26]. The experimental protocol was approved by the Institutional Committee for the Care and Use of Laboratory Animals at Cinvestav (CICUAL, Permit Number: 457–10). All efforts were made to minimize suffering.

The final sperm suspension was prepared as follows: for the tetracaine-caffeine assay, carried out at 25°C, it consisted in the following independent groups: untreated control sample, 8 mM tetracaine, 111 mM caffeine and the combination 8 mM tetracaine - 111 mM caffeine. For the tetracaine-calcium assay, carried out at 10°C (we explain later why the temperature is different) it consisted in the following independent groups: untreated control sample, 8 mM tetracaine, 200 mM CaCl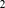 and the combination 8 mM tetracaine - 200 mM CaCl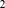. Drugs and CaCl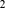 were purchased in Sigma-Aldrich. Each experiment was performed three times for a further statistical analysis. Tetracaine was previously disolved in dimethyl sulfoxide (DMSO) at 14 mM and it was verified that sperm motility is not affected by this compound.

### Liposomes preparation

Liposomes of 1,2-dipalmitoyl-sn-glycero-3-phosphocholine and dipalmitoyl phosphatidic acid (DPPC/DPPA) (Avanti Polar Lipids, Birmingham/AL) were prepared in a mole ratio of 95:5. The lipids were employed without further purification. DPPA was used to confer negative charge to the liposomes (at pH 7) and avoid flocculation. It is important to mention that the most abundant polyunsaturated lipid in mouse sperm plasma membrane [13] presents a melting transition below the minimum working temperature of our calorimeter (−10°C). Whereby, we choose to work with some of the most common lipids in plasma membranes with a melting transition above 0°C. First, appropriate amounts of the lipid stock solutions in chloroform were mixed to obtain the desired compositions. The resulting mixtures were then evaporated to dryness under a stream of nitrogen, keeping the mixture above its melting temperature (50°C), and forming a fully spread film at the bottom of a round bottom flask during the process. Second, the dried lipid film was hydrated with a buffer solution comprising the same ionic conditions used above (WH medium), and thereafter mixed in a vortexer. Next, the mixture was sonicated for 25 min in a water-bath ultrasonicator above melting temperature resulting in SUV liposomes.

### Calorimetry

Heat capacity profiles were recorded at a lipid concentration of 3 mg/ml (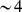
mM), with a heating rate of 1°C/min. Before they were loaded into precooled DSC cuvettes, the samples were degassed at low pressure during 10 min at 25°C. The calorimeter (Microcalorimeter, NanoDSC, TA Instruments) was interfaced to a PC, and data were analyzed using the software provided with the instrument. All samples were equilibrated during 8 min at 25°C. Three heating scans from 25°C to 50°C were performed for each sample. As in the sperm motility experiments explained above, same drugs and concentrations were used for the calorimetric analysis. Note that we added caffeine to samples that already had tetracaine and we added tetracaine to samples already doped with calcium. The order of addition does not affect the results.

Each experiment was performed only two times due to the robust results, namely, the calorimetric scans were reproducible (scan to scan and sample to sample) indicating that the final mixtures reached equilibrium. It was also verified that DMSO, at the working concentration (14 mM), does not change the phase behavior of the liposomes.

### Molecular Dynamics Simulations

The simulations ran 30 ns using GROMACS program with time steps of 2 fs. They were carried out using 200 DPPC molecules 15,000 water molecules, 10 caffeine and tetracaine molecules (five and five in the combination case) in a physiological medium at 37°C. Due to the low concentration in the experiments, DPPA is not used in the simulations. 3D structures, force fields and partial charges of the molecules, were obtained from different sources. The DPPC structure was obtained from [33], caffeine from [34], and tetracaine was built using the online server PRODRG (available at http://davapc1.bioch.dundee.ac.uk/prodrg/) and SwissParam (http://swissparam.ch/). In Figure 6 a cubic interpolation of order 10 was used to smooth the energy surfaces. While the time step to save the parameters of the simulation was 2 ps, each point in the trajectories depicted in Figure 7 was taken each 200 ps.
